# Pathophysiology and management of bleeding and thrombosis in patients with liver disease

**DOI:** 10.1111/ijlh.13856

**Published:** 2022-04-21

**Authors:** Bente P. van den Boom, Ton Lisman

**Affiliations:** ^1^ Surgical Research Laboratory and Section of Hepatobiliary Surgery and Liver Transplantation, Department of Surgery University of Groningen, University Medical Center Groningen Groningen the Netherlands

**Keywords:** bleeding, coagulation, haemostasis, liver disease, thrombosis

## Abstract

Patients with liver disease often develop complex changes in their haemostatic system. Frequently observed changes include thrombocytopaenia and altered plasma levels of most of the proteins involved in haemostasis. Although liver disease was historically classified as a haemostasis‐related bleeding disorder, it has now been well established that the antihaemostatic changes that promote bleeding are compensated for by prohaemostatic changes. Conventional coagulation tests however do not accurately reflect these prohaemostatic changes, resulting in an underestimation of haemostatic potential. Novel coagulation tests, such as viscoelastic tests (VETs) and thrombin generation assays (TGAs) better reflect the net result of the haemostatic changes in patients with liver disease, and demonstrate a new, “rebalanced” haemostatic status. Although rebalanced, this haemostatic status is more fragile than in patients without liver disease. Patients with liver disease are therefore not only at risk of bleeding but also at risk of thrombosis. Notably, however, many haemostatic complications in liver disease are not related to the haemostatic failure. It is, therefore, crucial to identify the cause of the bleed or thrombotic complication in order to provide adequate treatment. In this paper, we will elaborate on the haemostatic changes that occur in liver disease, reflect on laboratory and clinical studies over the last few years, and explore the pathophysiologies of bleeding and thrombosis in this specific patient group.

## 1 INTRODUCTION

The liver plays a central role in the activation and regulation of the haemostatic system as it is the site of the synthesis of many haemostatic proteins. The development of liver disease therefore understandably may affect the haemostatic balance substantially. Advanced liver disease is characterized by complex alterations of both primary and secondary haemostasis and fibrinolysis.^1^ Historically, the antithrombotic changes in liver disease, such as thrombocytopaenia and low levels of coagulation and fibrinolytic proteins, have led to the notion that patients with cirrhosis were at high risk of bleeding, and potentially even “auto‐anticoagulated” and thus protected from thrombotic disease. Abnormal conventional laboratory coagulation test results—such as platelet counts, the prothrombin time, and the activated partial thromboplastin time—supported these hypotheses. Nowadays, it has, however, been well established that the antithrombotic changes that occur in patients with liver disease are compensated for by prothrombotic changes that occur simultaneously. The net result of both antithrombotic and prothrombotic changes is a new, but fragile haemostatic “rebalance,” with notable hypo‐ and hypercoagulable features.^1^ This new balance can, however, easily be disrupted, for instance by infection or decompensation of disease. Consequently, patients with cirrhosis are at increased risk of both thrombosis and bleeding. However, many haemostatic complications that occur in these patients cannot be directly attributed to haemostatic changes, and the underlying pathogenesis of a bleed or thrombotic episode likely determines the best therapeutic target.

Although the concept of “rebalanced” haemostasis has now been widely accepted by both the hepatology and haematology communities,^2^, ^3^ we are not yet able to accurately predict and prevent haemostatic complications in this heterogenous patient group. Also, due to the paucity of clinical data optimal strategies to prevent or treat haemostatic complications have not been established. We are, however, slowly but steadily gaining new insights into the pathogenesis of haemostatic complications in patients with liver disease. In this review, we will elaborate on the complex alterations that might occur in patients with liver disease, and reflect on lessons learned from recent laboratory and clinical studies.

## 2 HAEMOSTATIC CHANGES IN LIVER DISEASE

**FIGURE 1:**
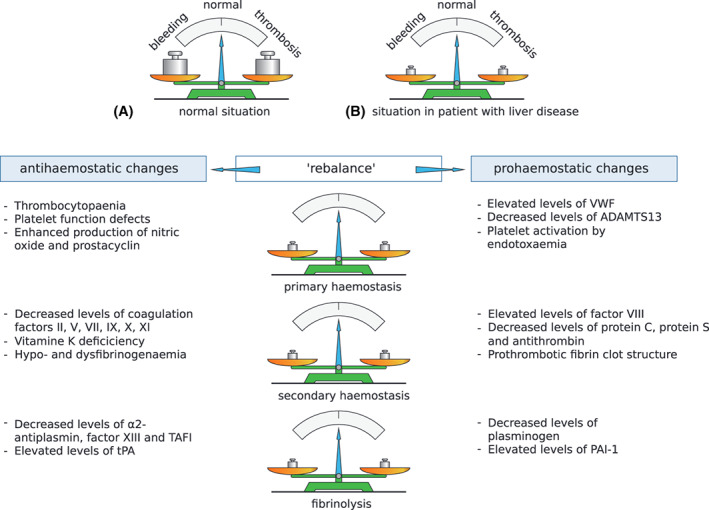
Schematic presentation of the rebalanced haemostasis in patients with liver disease. In healthy individuals (A), haemostasis is in solid balance. In patients with liver disease (B and table) both pro‐ and antihaemostatic changes result in a “rebalance” of the haemostatic system. This new balance is however more fragile, and may therefore more easily tip towards bleeding or thrombosis. Abbreviations: ADAMTS13, a disintegrin and metalloproteinase with a thrombospondin type 1 motif, member 13; PAI‐1, plasminogen activator inhibitor‐1; TAFI, thrombin activatable fibrinolysis inhibitor; tPA, Tissue plasminogen activator; VWF, von Willebrand factor. Modified from Warnaar et al.^4^ with permission from Wolters Kluwer Health

An overview of the haemostatic changes that occur in patients with liver disease is depicted in Figure 1. Even though there are many similarities in the haemostatic changes that occur in acute and chronic liver disease, there are some distinct features that characterize each. For example, in acute liver failure thrombocytopaenia is typically milder than in chronic liver disease, whereas plasma levels of coagulation factors are usually lower in acute liver failure.^5^ In chronic liver disease, haemostatic profiles appear similar among various aetiologies,^6^ although the cholestatic liver disease has also been associated with a more hypercoagulable profile compared to other aetiologies.^7^

### 2.1 Changes in primary haemostasis

#### 2.1.1 Thrombocytopaenia

Thrombocytopaenia is the most common haematological abnormality found in patients with chronic liver disease,^8^ occurring in up to 78% of patients with cirrhosis. Although the prevalence of thrombocytopaenia is relatively high in this patient group, the majority (86%) of these patients only experience mild thrombocytopenia (platelet count <150 × 10^9^/L).^9^ More severe stages, such as moderate (platelet count <100 × 10^9^/L) and severe thrombocytopaenia (platelet count <50 × 10^9^/L), occur in 13% and 1% of patients with cirrhosis, respectively.^9^

The cause of thrombocytopaenia in chronic liver disease has long been attributed to sequestration by the spleen, due to congestive splenomegaly secondary to portal hypertension.^10^ The concept of congestive splenomegaly being the sole cause of thrombocytopaenia however seems contradictory, as not all cirrhotic patients with thrombocytopaenia have splenomegaly, and as treatment of splenomegaly (e.g. by reducing portal pressure) does not necessarily lead to normalized platelet numbers. Although sequestration by the spleen certainly attributes to the low platelet counts in patients with liver disease, other factors—such as reduced production and increased destruction of platelets—contribute to the development of thrombocytopaenia.

Both reduced thrombopoietin synthesis and bone marrow suppression may contribute to reduced platelet production in patients with liver disease. As thrombopoietin is mainly synthesized by hepatic parenchymal and sinusoidal cells, patients with a decreased synthetic capacity of the liver often have low levels of serum thrombopoietin,^11^ and levels rapidly normalize following successful liver transplantation.^12^ Indeed, therapeutic agents that simulate thrombopoietin function, such as thrombopoietin receptor agonists (TPO‐RA) eltrombopag, avatrombopag and lusutrombopag, have shown to be effective in increasing platelet numbers in patients with cirrhosis.^13^ Viruses, alcohol, iron overload, and medications may result in bone marrow suppression leading to a decreased production of platelets.^9^ Although hepatitis C is most notorious for causing thrombocytopaenia (in part induced by bone marrow suppression, among other mechanisms), both hepatitis A and B may also lead to bone marrow suppression.^14^ Alcohol use, iron overload and medications such as azathioprine, antibiotics and interferon might contribute to dysfunctional megakaryopoiesis, and may, therefore, contribute to the thrombocytopaenia of chronic liver disease. Finally, platelet counts may be affected by increased platelet destruction—for example, caused by (systemic) activation of platelets or immune‐mediated destruction.^9^

#### 2.1.2 Platelet function

Even though there is evidence of an altered platelet function in patients with chronic liver disease, platelet function in these patients remains poorly characterized. Importantly, studies have been highly conflicting with some denoting platelets function defects, whereas other studies have demonstrated normal or even increased platelet function.^15^ Early in vitro studies using light transmission aggregometry (LTA) show markedly reduced aggregation of platelets of patients with cirrhosis after stimulation with adenosine diphosphate (ADP), arachidonic acid, collagen and thrombin.^9^ Notably, LTA test results are unreliable at low platelets counts, and the thrombocytopaenia of liver disease needs to be taken into account when performing platelet function tests. Although some studies have adjusted platelet counts in patient and control samples to a certain level to allow adequate comparisons, this strategy might be suboptimal as samples need to be manipulated prior to measurement, introducing possible bias. Alternatively, platelet function studies that are not sensitive to platelet counts (such as flow cytometry) could be performed. Recent studies that take the thrombocytopaenia of liver disease into account, however, are still contradictory.^15^, ^16^ Thus, to what extent platelet function is altered in patients with the liver disease remains uncertain. Moreover, whether a possibly altered platelet function has any clinical relevance is debatable, and is subject to future research.

#### 2.1.3 Von Willebrand factor and ADAMTS13

The antithrombotic effects of thrombocytopaenia and potentially decreased platelet function are (at least partially) compensated by prothrombotic changes, such as increased plasma levels of von Willebrand factor (VWF) and decreased levels of its regulator ADAMTS13.^17^ Previous studies by our group and by others have not only shown that patients with chronic liver disease have elevated levels of VWF, but also that levels increase proportionally to disease severity with mean levels of around 300% in stable cirrhosis, 360% in acutely decompensated cirrhosis and 700% in acute‐on‐chronic liver failure (ACLF).^18^ Similarly, mean levels of ADAMTS13 decrease to around 90%.^18^ Surprisingly, despite low ADAMTS13 levels, the multimeric size of the VWF molecule seems to be reduced, which has been attributed to VWF proteolysis by other proteases such as plasmin and elastase.^19^ There is increasing evidence that this VWF:ADAMTS13 imbalance compensates for decreased platelet levels and/or function: despite thrombocytopaenia and low haematocrit, platelet adhesion of patients with liver disease appears to be similar to that of healthy controls in in vitro models of platelet adhesion under flow conditions.^17^, ^19^

In liver disease, endothelial dysfunction is a major determinant in the development of portal hypertension.^20^ Recent studies have shown that elevated VWF levels indeed correlate with increased portal pressure, and might even serve as a non‐invasive marker of portal hypertension.^21^ Interestingly, VWF levels do not only predict variceal bleeding,^22^ but also decompensation of disease and mortality^21^ in patients with chronic liver disease. Another study indicates that the combination of VWF levels and Model for End‐stage Liver Disease (MELD) scores better stratifies the risk of mortality than MELD scores alone.^23^ The results of these studies look promising, and further research is indicated to determine potential clinical applications of VWF measurements in patients with liver disease.

### 2.2 Changes in secondary haemostasis

Hepatocytes are responsible for the production of nearly all plasma proteins involved in fibrin formation, with the exception of factor VIII and tissue factor pathway inhibitor. In patients with liver disease, in which the synthetic function of the liver is impaired, low plasma levels of clotting factors such as factors V, VII, IX, X, XI, and prothrombin are often observed.^1^ Even though fibrinogen levels are within the normal range in stable liver disease, low levels of fibrinogen are typically found in more advanced liver disease.^18^ Conversely, levels of factor VIII are often elevated, possibly due to upregulation of FVIII synthesis by endothelial cells, elevated levels of its carrier protein VWF, or impaired clearance by the liver.^24^ The deficiency of the procoagulant clotting proteins is, at least in part, compensated for by decreased levels of anticoagulant proteins such as protein C, protein S, antithrombin, heparin co‐factor II, and α2‐macroglobulin. These natural antihaemostatic proteins are all synthesized by the liver, and low levels of these proteins are often found in patients with liver disease.^1^

Conventional coagulation tests, such as the prothrombin time (PT, and the PT‐derived International Normalized Ratio, INR) and the activated partial thromboplastin time (aPTT), do not reflect coagulation status in patients with liver disease. Even though these coagulation tests accurately reflect changes in prohaemostatic pathways, they are insensitive to the regulators of these pathways, i.e. antithrombin, protein C and protein S. These tests, therefore, present a one‐sided view, and do not take the complex alterations in the antihaemostatic pathways of patients with liver diseases into account.

Alternative tests for assessing coagulation status include viscoelastic tests (VETs) and thrombin generation assays (TGA). Such tests are sensitive to part of the natural anticoagulant mechanisms, and thereby better represent the coagulation capacity of patients with changes in both pro‐ and anticoagulant pathways. VETs, such as thromboelastography (TEG) and thromboelastometry (ROTEM), are point‐of‐care tests that measure the kinetics of a forming blood clot over time. In accordance with the concept of rebalanced haemostasis, VET results are often normal in patients with chronic liver disease.^7^ Additionally, VET parameters do not correlate with conventional coagulation tests, such as the PT/INR. However, some studies, in particular those using ROTEM, show a hypocoagulable profile in patients with chronic liver disease.^25^, ^26^ These unambiguous results may be explained by the variation in reagents used to activate coagulation. Clotting times in ROTEM analyses are shorter than those in TEG assays due to the use of more potent reagents in ROTEM studies. This “quick start” in ROTEM in part overwhelms the antihaemostatic responses and therefore ROTEM is less sensitive to antihaemostatic proteins than TEG.^27^ Which of these two VETs gives a better representation of the haemostatic status in patients with the liver disease remains unclear. Moreover, although these VETs have clear advantages over conventional coagulation tests, they still do not fully represent haemostatic balance. Of note, both TEG and ROTEM are insensitive to the antihaemostatic protein C‐system (which requires activation by endothelial cell transmembrane protein thrombomodulin) and VWF.^27^ These VETs may, therefore, substantially underestimate the haemostatic status in patients with liver disease, in which changes in protein C and VWF are frequently observed.^1^ Even though VET‐based transfusion algorithms are gaining in popularity, sufficient evidence that these lead to an optimal approach to transfusion is currently lacking. Its values in the prediction of (periprocedural) bleeding (or thrombosis) are debatable: a recent pilot study indicates no clear correlation between a hypocoagulable status as determined by ROTEM and the occurrence of bleeding.^28^ Large prospective studies are necessary to determine predictive values of VETs, and until then, caution should be taken in the interpretation of abnormal VET results.

Global coagulation status is also frequently assessed by TGAs. In studies on patients with liver disease, TGAs modified by the addition of thrombomodulin or other activators of the protein C system have been frequently used.^29^ Using TGAs that are sensitive to changes in the protein C system, a more honest representation of the haemostatic balance is obtained when compared to VETs. Interestingly, most studies using modified TGA show normal to enhanced thrombin generating capacity even in the sickest patients with exceptionally low factor levels. However, similar to VETs, studies did not find a correlation between the haemostatic status as assessed by TGA^28^ and the occurrence of bleeding, and large‐scale studies are necessary to evaluate its role in the prediction of bleeding and thrombosis. TGAs are generally limited by the use of plasma, dismissing the role of red and white blood cells and platelets in coagulation. One study has used TM‐modified whole blood TGA to study the haemostatic status of patients with liver disease,^30^ but this technology requires further optimization.

### 2.3 Changes in fibrinolysis

Apart from tissue‐type plasminogen activator (tPA) and plasminogen activator inhibitor‐1 (PAI‐1), all proteins involved in fibrinolysis are synthesized by the liver. As anticipated, reduced levels of plasminogen, plasmin inhibitor, thrombin activatable fibrinolysis inhibitor (TAFI), and factor XIII are found in both acute and chronic liver failure.^1^ In contrast, plasma levels of the profibrinolytic protein tPA and antifibrinolytic protein PAI‐1 are often increased, likely due to enhanced endothelial cell activation or a reduced clearance.

Historically, patients with liver disease were considered to be hyperfibrinolytic. More recent studies, however, using plasma‐based clot lysis assays have indicated a fibrinolytic “rebalance” in patients with stable liver disease due to a simultaneous decline in pro‐ and antifibrinolytic proteins.^31^ Patients with very advanced chronic liver disease, however, appear to have altered fibrinolytic profiles, with a subset of patients that are clearly hyperfibrinolytic and a subset with clear hypofibrinolysis.^32^ Patients with a hypofibrinolytic profile often have sepsis, and it is well known that sepsis without underlying liver disease is associated with a hypofibrinolytic state. In patients with acute liver failure, there is a uniform and severe hypofibrinolytic state, likely related to highly elevated plasma PAI‐1 levels.

## 3 CLINICAL EVIDENCE OF HAEMOSTATIC REBALANCE

The concept of rebalanced haemostasis which is demonstrated by the aforementioned laboratory findings, is supported by clinical evidence. A clear example of the compensation of prohaemostatic defects by antithrombotic changes is that the INR does not correlate with the occurrence of bleeding.^33^ Another example is the occurrence of bleeding during major surgical procedures, such as orthotopic liver transplantation. When orthotopic liver transplantation first became available as a treatment for end‐stage liver disease in the 1970s, major periprocedural bleeding occurred frequently. Blood transfusion requirements of 20 to 40 units of red blood cell concentrates, platelet concentrates and plasma to counteract the enormous amount of blood loss were therefore not uncommon.^34^ Over the past two decades, transfusion requirements have however dramatically decreased, and an increasing amount of transplantation centres report to be able to safely perform transfusion‐free transplants—sometimes in up to 77.4% of all transplants^35^! Now, if cirrhosis would indeed lead to a “true” antihaemostatic state, as is, for example, the case in haemophilia, major surgical procedures without prophylactic prohaemostatic treatment would likely lead to disastrous bleeding. A similar phenomenon can be observed in studies on periprocedural bleeding in low‐risk procedures. Despite severe thrombocytopaenia (platelet count <50 × 10^9^/L) or prolonged coagulation times (INR >1.5), procedures such as paracentesis and thoracocentesis can be performed safely, without correction of hemostasis.^36^ There appears to be a paradox with regards to bleeding complications in patients with liver disease: even though these clinical examples underline the overestimation of the risk of bleeding caused by haemostatic imbalance, haemorrhagic complications remain the most prevalent “haemostatic” complication in this patient group. To better understand this apparent paradox, it is important to distinguish the various pathophysiologies of bleeding in patients with liver disease.

## 4 HAEMOSTATIC COMPLICATIONS IN LIVER DISEASE

### 4.1 Bleeding

**FIGURE 2:**
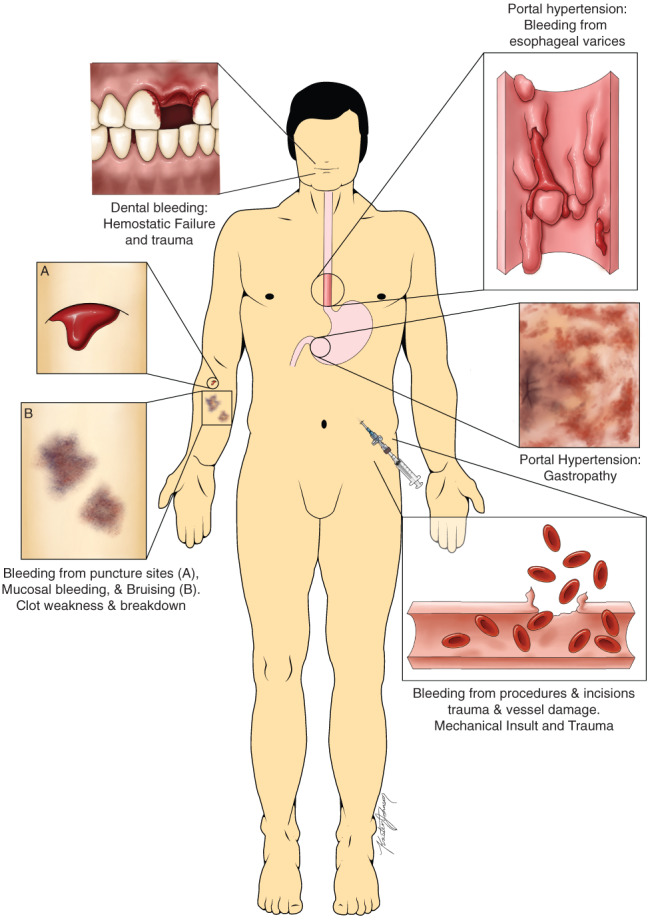
Depiction of various types of bleeding that a patient with cirrhosis might experience. Some bleeding sources are related to vascular trauma, some to excessive portal hypertension, and some to haemostatic failure. Other common sources not shown include portal hypertensive enteropathy/colopathy, rectal varices (portal hypertensive), epistaxis (mechanical or haemostatic), and menorrhagia (haemostatic). Figure from Northup et al., used with permission^2^

Bleeding is the most common haemostatic complication in patients with liver disease, but not all bleeding is caused by disruption of the haemostatic balance. A recent guidance document from the American Association for the Study of Liver Diseases (AASLD) defines three distinct pathophysiologies of bleeding^2^: bleeding due to portal hypertension (e.g. variceal bleeding), haemostatic failure, or (mechanical) vascular trauma (Figure 2). Importantly, all three components may play a role in procedure‐related bleeding, resulting in complex dilemmas for clinicians as to how to prevent and treat these bleeds.

Variceal bleeding occurs in 25%–35% of patients with cirrhosis and accounts for up to 90% of all haemorrhagic complications.^37^ Acute variceal bleeding is a life‐threatening situation with a mortality of 15%–30% at first bleed, making it the number one cause of death among patients with cirrhosis.^38^ It has been widely accepted that the presence and rupture of oesophageal varices is a consequence of portal hypertension rather than haemostatic failure.^38^ Risk factors for variceal bleeding thus include signs of portal hypertension, such as the presence of large varices and the presence of *red wale marks*, as well as severe cirrhosis (Child Pugh C).^39^ The fact that low‐molecular‐weight heparins (LMWH) do not aggravate the variceal bleed,^40^ and the observation that prohaemostatic treatment with recombinant factor VIIa does not reduce bleeding is evidence for the absence of a haemostatic component in variceal bleeding.^41^ Treatment of variceal bleeding focuses on resuscitation of the patient (with the restoration of the mean arterial pressure, MAP), reduction of portal pressure, and endoscopic banding or ligation of the varices. Although red blood cell concentrates and fluid restoration may be crucial in the management of major bleeding, a restrictive transfusion strategy is recommended, as portal pressure is directly correlated to MAP. Overcorrection of haemoglobin or administration of fresh frozen plasma (FFP) or platelet concentrate may thus promote bleeding by inducing fluid overload and should be avoided. Notably, transfusion of prohaemostatic agents, such as FFP, is explicitly not indicated.^2^ A recent study has demonstrated that FFP has a notably unfavourably risk‐benefit ratio in variceal bleeding^42^: not only was treatment with FFP associated with failure to control bleeding at 5 days, it was also independently associated with mortality at 42 days. Similarly, treatment with the antifibrinolytic agent tranexamic acid is not only ineffective in the cessation of variceal bleeding, it was also associated with an increased risk of venous thromboembolic events.^43^

Although portal pressure‐related (i.e. variceal) bleeds are by far the most common bleed in patients with chronic liver disease, haemostatic failure‐related bleeds also occur. Spontaneous, haemostatic failure‐related bleeds include epistaxis, gum bleeds, menometrorrhagia, purpuric skin lesions and prolonged bleeding after venepuncture or insertion of central lines. Albeit frequently occurring and possibly causing discomfort, these bleeds seldomly pose risk to life. Treatment of these bleeds varies among bleeding sites but often consists of compression or other local measures. As most of these bleeds are minor, blood product transfusion is rarely needed. In the case of major haemostatic bleeding, it may be difficult to assess what prohaemostatic therapy is most suitable, as bleeding may be caused by either primary or secondary haemostatic defects or a hyperfibrinolytic status. In these cases, VETs may help in the decision‐making process. A recent RCT showed that a TEG‐guided transfusion strategy leads to a significantly lower use of blood components compared with the current standard of care strategies (guided by INR and platelet count) in patients with cirrhosis with acute non‐variceal upper gastrointestinal bleeding.^44^ Moreover, this TEG‐guided transfusion strategy did not lead to an increase in failure to control bleeding, failure to prevent rebleed, and mortality. Although this first RCT showed promising results with lower transfusion requirements, convincing evidence on the actual prognostic value of TEG in assessing bleeding risk (and the concomitant cut‐off values that should be applied) is currently still lacking. Its results should, therefore, be interpreted cautiously and studies in which a “no transfusion arm” is included should be performed.

The risk of periprocedural bleeding depends on various factors, such as the type of procedure and technical skills of the operator, liver disease‐related factors (portal pressure, haemostatic status), and systemic factors (infection, renal disease). Procedure bleeding risk has typically been categorized as low‐risk (<1.5% risk of major bleeding, easy to control) and high risk (>1.5% risk of major bleeding, possibly difficult to control).^2^ The most commonly performed procedures in patients with chronic liver disease are associated with a low bleeding risk (paracentesis, thoracocentesis, central venous catheter placement, oesophagogastroscopy + − variceal band ligation, bronchoscopy) rather than high bleeding risk (biliary interventions, liver biopsy, TIPS, ERCP, central nervous system procedures, intra‐articular injections, dental extractions). Indeed, the prevalence of periprocedural bleeding during these procedures is low: studies show clinically significant bleeding rates of 0.2%–0.4% in paracentesis and thoracocentesis, <0.2% in central venous catheterisation, 0.2%–0.6% in liver biopsy, and 1.2%–7.3% in endoscopic variceal band ligation.^36^ Although one study reported excess bleeding in 12.6% of dental extractions, which is seemingly high, all of these bleeds could be controlled using only local measures.^45^ Interestingly, prolongation of INR and thrombocytopaenia did not predict bleeding outcomes in the majority of these studies, and of note, even in patients with platelet counts below 20 × 10^10^/L, low‐risk procedures such as para‐ and thoracocentesis could be performed safely. In patients with acute liver failure—in whom portal hypertension and oesophageal varices are rare, but haemostatic abnormalities can be profound—clinically significant bleeding is very uncommon.^46^

Regardless of the low bleeding risk in patients with liver disease, clinicians frequently prescribe prophylactic transfusion of prohaemostatic agents prior to invasive procedures. Protocols for prohaemostatic prophylaxis are based on the general population, and often advise prophylactic correction in case of elevated INR or severe thrombocytopaenia. A clear example of these protocols is the transfusion guidelines of the American Society of Haematology, which advise transfusion of platelets in patients with platelet levels of <50 × 10^9^/L and transfusion of FFP in patients with an INR of >2.0 (>1.5 in neurosurgical patients) prior to invasive procedures.^47^ Cut‐off values of INR and platelet count may, however, differ among countries, or even among hospitals or medical specialities within hospitals. The frequent abnormalities in INR and platelet levels in patients with chronic liver disease continue to lead to a perceived high risk of bleeding in these patients, whereas substantial evidence against a relation between abnormal conventional coagulation tests and the risk of bleeding in these patients exists. Prophylactic blood product transfusions are thus frequently prescribed in patients with chronic liver disease, with rates of 11.7% in a nationwide UK study,^48^ to up to 33.6% in patients at a tertiary liver care centre in India.^49^ Data on the efficacy of FFP transfusions in correcting haemostatic abnormalities are contradictory: in one study, FFP transfusion led to a minimal increase in thrombin formation (5% increase in ETP),^50^ whereas in a recent study by our group this increase was more profound (20% increase in ETP).^51^ Of note, in the latter study, baseline ETP in patients with chronic liver disease was similar to that in healthy controls, raising the question of whether transfusion of FFP was actually indicated. More importantly, however, there is currently no high‐level evidence that prophylactic FFP transfusions prevent bleeding in patients with coagulopathy, regardless of whether this coagulopathy is caused by liver disease.^52^ As transfusion of blood products may also lead to adverse events—such as allergic reactions, transfusion‐related circulatory overload (possibly even promoting bleeding), transfusion‐related acute lung injury (TRALI), transfusion‐transmitted infections and graft versus host disease—current guidance documents strongly advise against prophylactic use of FFP in patients with chronic liver disease.^2^, ^53^, ^54^ Although (very) low levels of fibrinogen appear to be predictors of major bleeding in critically ill patients with cirrhosis,^55^ transfusion of cryoprecipitate does not affect survival or bleeding complication in these patients.^56^ Low levels of fibrinogen may therefore be more indicative of decreased hepatic synthesis rather than directly involved in the development of a bleeding episode. The requirement for interventions directed at elevating the platelet count is more controversial. Although some researchers argue that platelet transfusions may be helpful in the prevention of bleeding in high‐risk invasive interventions, there is insufficient data that supports this statement. In addition, (low‐risk) invasive interventions can be performed safely in patients with chronic liver disease and low platelets, without the use of prophylactic platelet transfusion.^36^ Over the last decades, TPO‐RAs have gained popularity in the correction of platelet levels prior to planned invasive procedures. Although initial studies using eltrombopag were associated with an elevated risk for thrombosis, more recent clinical trials show that TPO‐RAs avatrombopag and lusutrombopag are indeed effective in increasing platelet levels, lead to less platelet transfusions, and lead to less periprocedural bleeding without raising the risk of thrombosis in patients with liver disease.^13^ However, these bleeding complications included clinically non‐significant bleeds and bleeds that occurred prior to the procedure, which questions the need for correction of the platelet count. TPO‐RAs may be associated with adverse reactions, and can only be used in planned procedures, as it will take a few weeks to reach normalized platelet levels. Although it is still unclear whether correction of platelet count has any benefit, TPO‐RAs may be preferable to platelet transfusions due to the lack of transfusion‐related adverse reactions.

### 4.2 Thrombosis

Hospitalized patients with cirrhosis have a twofold greater risk of venous thromboembolism (VTE) compared to patients without cirrhosis. The estimated incidence of VTE in patients with cirrhosis is 0.5%–6.3%,^57^ and although thrombosis risk may be proportional to the severity of disease,^58^ little is known about specific risk factors for VTE in patients with liver disease. Optimal treatment strategies for VTE in patients with chronic liver disease have not yet been established. Until more data become available on the optimal anticoagulant agents and their dosages, guidelines for thrombosis in the general population should likely best be followed.^59^ This approach may, however, lead to some complex issues, such as how to accurately monitor the efficacy of vitamin K antagonists (VKA). In the general population, VKA monitoring is based on the INR. Due to an increased INR at baseline in patients with advanced chronic liver disease, this target range may however be inadequate for these patients. Novel treatment strategies, such as direct oral anticoagulants (DOACs), bypass the dosing issues of VKAs. Unfortunately, however, patients with chronic liver disease were excluded from phase III trials, resulting in insufficient data on the efficacy and safety of these agents in this patient group. Moreover, nearly all of these DOACs are metabolized and/or cleared by the liver, and cirrhosis may therefore affect plasma medication levels or lead to accumulation. Although a recent pilot study did not show any apparent accumulation of edoxaban in patients with Child‐Pugh A cirrhosis,^60^ randomized clinical trials that support these findings are lacking. In patients with more severe chronic liver disease, treatment of VTE with (low‐molecular‐weight) heparins may therefore be the safest alternative.

Conventional coagulation tests do not predict thrombosis, and an elevated INR does explicitly not protect against VTE in patients with chronic liver disease. However, due to abnormal conventional coagulation tests, prophylactic anticoagulation is often withheld from patients with the chronic liver disease upon hospitalization. This may in part be caused by the historical association of liver disease and life‐threatening bleeding events (and thus the fear of provoking such an event), or by the lack of (internationally) recognized prediction measures and guidelines that take the complex haemostatic alterations in liver disease into account. Retrospective studies indicate that VTE prophylaxis is safe and does not increase bleeding rates or severity of bleeding in patients with chronic liver disease.^59^ Interestingly, however, VTE prophylaxis also does not seem to decrease VTE rates in these retrospective studies, suggesting that the prophylactic regimens administered are suboptimal. Although in vitro studies have suggested a decreased potency of some anticoagulant drugs in patients with liver disease,^61^ in a recent prospective study, a single dose of prophylactic heparins led to a similar decrease in thrombin generation in patients with chronic liver disease compared to patients without liver disease.^62^ Although future studies on the prolonged use of VTE prophylaxis and its optimal dosages should be performed, VTE prophylaxis should not be withheld from patients with cirrhosis. Similar to the treatment of VTE, guidelines on VTE prophylaxis for the general population should be followed.^2^

**FIGURE 3:**
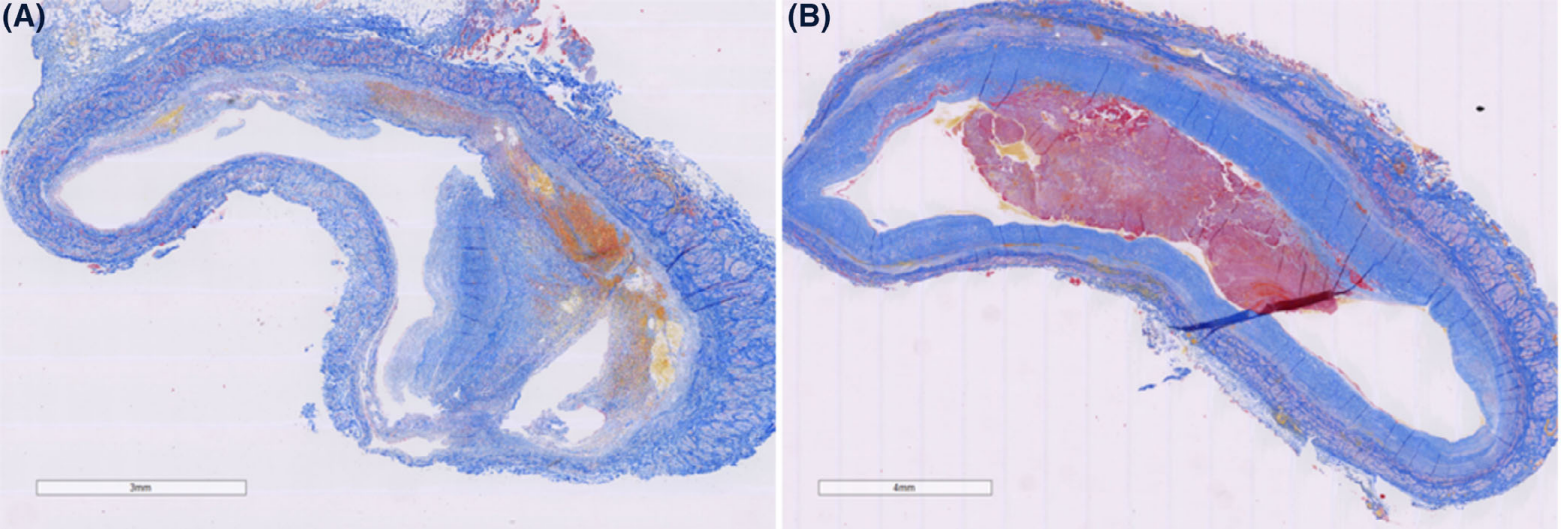
Martius scarlet blue (MSB)‐stained sections of extrahepatic portal vein samples removed during liver transplantation. (A) A portal vein thrombus consisting of a focally thickened intimal layer of the vessel wall with some haemorrhage but without a fibrin‐rich thrombus. (B) A Portal vein thrombus consisting of a circumferential thickened intimal layer of the vessel wall with a fibrin‐rich thrombus on top. Figure from Driever et al., used with permission.^64^

Portal vein thrombosis (PVT) is a type of VTE that rarely occurs in the general population but is frequently observed in patients with chronic liver disease. Reports on the incidence of PVT in patients with the chronic liver disease vary from 0.6% to 26%.^57^ The prevalence increases with the severity of the disease, with a prevalence of 1% in patients with the compensated disease, to 8%–25% in liver transplantation candidates. These rates may however underestimate the actual rates of PVT, as PVTs are often asymptomatic and the sensitivity among diagnostic methods (ultrasound, CT, MRI) varies.^57^ The pathogenesis of PVT is not fully understood. Previous retrospective studies have suggested hypercoagulability to be a risk factor for PVT development. However, a recent large and prospective study suggests that not hypercoagulability, but the severity of disease and portal flow are important risk factors for the development of PVT.^63^ A recent study by our group showed that PVT in patients with cirrhosis frequently does not consist of a true ‘thrombus’ as it often lacks fibrin and platelets. Instead, PVT was consistently found to consist of intimal fibrosis in the portal vein wall. In only one‐third of thrombi, a fibrin‐rich thrombus was present on top of this thicked intimal lesion (Figure 3).^64^ This observation thus also questions the central role of the haemostatic system in PVT development and may explain why many patients with PVT do not respond to anticoagulant treatment.^64^ Guidelines by the AASLD recommend anticoagulant therapy in patients with cirrhosis with recent (partially) occlusive thrombosis in the main portal vein or mesenteric veins, to avoid thrombosis progression that might hinder liver transplantation or cause progression of portal hypertension.^2^ Although anticoagulant therapy may promote recanalization of the portal vein in acute PVT specifically,^65^ there is no established benefit of anticoagulant therapy in chronic complete occlusion of the portal vein. Data on the optimal treatment and prevention of PVT is, however, scarce, and future studies should be performed to assess the role of (lifelong) anticoagulant therapy and other modalities in the management of PVT in patients with chronic liver disease.

## 5 CONCLUSION

The haemostatic alterations that occur in chronic liver disease are complex and lead to a net result of a new, but fragile rebalanced haemostasis. Conventional coagulation tests do not predict haemostatic complications in patients with chronic liver disease, and convincing evidence on the predictive value of VETs and TGA is currently still lacking. Although bleeding and thrombosis may occur in patients with liver disease, not all haemostatic complications can be ascribed to haemostatic failure. Treatment with anticoagulant agents appears safe, and VTE prophylaxis should not be withheld from these patients although optimal strategies with regard to dosing should be explored. Conversely, FFP transfusions should not be given to patients with chronic liver disease prior to (low‐risk) invasive procedures or acute bleeding, as they likely do not decrease and possibly even increase the risk of bleeding. The role of platelet transfusions or treatment with TPO‐RAs as prophylaxis prior to invasive procedures is controversial and should be explored further. Large‐scale clinical research is direly needed to formulate clear clinical guidelines on the management of bleeding and thrombosis in patients with chronic liver disease.

## CONFLICT OF INTEREST

The authors declare no competing interests.

## Data Availability

Data sharing not applicable to this article as no datasets were generated or analysed during the current study.
